# Multi-Source Evidence Chain Identification and Mechanistic Analysis of a Creep-Type Expressway Embankment Landslide in a Mountainous Karst Area: A Case Study of the K610 Section of the Rucheng-Chenzhou Expressway

**DOI:** 10.3390/s26196136

**Published:** 2026-09-28

**Authors:** Bin Liu, Fan Xu, Menggang Yang

**Affiliations:** 1 School of Civil Engineering, Central South University, Changsha 410075, China; mgyang@csu.edu.cn; 2 Hunan Provincial Communications Planning, Survey and Design Institute, Changsha 410200, China; 3 Key Laboratory of Highway Construction and Maintenance Technology in South Central China, Changsha 410200, China

**Keywords:** expressway embankment landslide, InSAR, strength reduction method, creep deformation, multi-source evidence chain

## Abstract

Recurrent settlement of high-fill embankments along mountain expressways is difficult to diagnose because its underlying mechanism is often unclear. This study investigates the distressed K610 section of the G76 Xiamen–Chengdu Expressway between Rucheng and Chenzhou by integrating time-series InSAR, ground-settlement monitoring, boreholes, electrical resistivity imaging, ground-penetrating radar, hydrochemistry, engineering records, and three-dimensional finite-element analysis. These data establish a multi-source evidence chain linking deformation, geological structure, hydrological processes, and mechanical response. InSAR-derived line-of-sight velocities range from −30 to −8 mm/yr, and representative points show cumulative displacements of −40 to −50 mm. The anomalous zone extends from the high-fill embankment into the natural slope and spatially coincides with observed settlement, the inferred sliding direction, and radar-detected voids. Groundwater and karst cavities were encountered in 14 and 11 of 39 boreholes, respectively, while three resistivity profiles revealed low-resistivity anomalies beneath the core distressed section, indicating the control of shallow groundwater and karst structures. Water samples from the distressed zone contained an average fluoride concentration of 0.59 mg/L, far below the 17.54 mg/L in the tailings reservoir and similar to mountain surface water, identifying rainfall infiltration as the dominant recharge pathway. Strength-reduction analysis yielded factors of safety of 1.12 under natural conditions and 1.01 under extreme rainfall, a 9.8% decrease; simulated shear-strain concentration zones corresponded closely to field distress. Collectively, the evidence supports a progressive mechanism of rainfall infiltration, water-induced weakening, slope creep, and differential embankment settlement. The framework distinguishes slope-scale creep from localized settlement and supports treatment-boundary delineation and remediation design.

## 1. Introduction

As linear transportation infrastructure, mountain expressways commonly traverse complex geological settings such as steep slopes, valleys, karst depressions, and cut-fill transitions. In these settings, embankment fills are strongly coupled with the underlying natural slopes, groundwater seepage systems, and karst conduits. Hungr et al. [1] updated the Varnes classification of landslides and noted that many slow-moving slides and complex landslides become apparent only during infrastructure operation; their failure modes often exhibit time-dependent creep, softening, and progressive propagation. Leroueil [2] systematically reviewed the movement and failure mechanisms of natural and cut slopes and emphasized the importance of the creep-deformation stage over engineering timescales. For expressway embankments constructed on active or potentially unstable slopes, pavement subsidence, longitudinal cracking, compression-induced failure of drainage facilities, and damage to slope-protection frames may merely be surface manifestations of persistent deformation within a deeper coupled slope-embankment system. Pavement leveling or localized embankment reinforcement may temporarily restore traffic serviceability but cannot reveal the root cause of recurrent distress [3,4,5]. Slow-moving landslides can persist for years while accumulating damage to roads and other infrastructure [6,7].

Satellite synthetic aperture radar interferometry (InSAR) has become an important technique for detecting slow-moving landslides and infrastructure deformation. PSInSAR, SBAS, SqueeSAR, and StaMPS established the methodological foundations for stable-scatterer analysis, small-baseline subsets, and multitemporal inversion [8,9,10,11]. Recent reviews further indicate that the principal advantage of InSAR is not to replace geological investigation but to provide long-term deformation monitoring at the slope scale [12,13]. For expressway slopes and embankment distress, previous studies have combined InSAR with GNSS, geophysical exploration, or numerical simulation to identify activity, assess risk, and prioritize remediation [14,15]. Permanent-scatterer observations and multi-year radar records show that slow-moving landslide velocities can vary with rainfall [16]. For recurrent embankment distress, a remaining task is to connect these observations to site-specific subsurface conditions and treatment history.

Expressway embankment distress in karst areas has distinctive characteristics. Karst collapse, settlement of the overburden, and groundwater seepage erosion are typical geological hazards affecting infrastructure in karst terrain [17]. Engineering assessment of karst sites must simultaneously consider the spatial heterogeneity of cavities, variations in overburden thickness, and fluctuations in groundwater level [18,19]. InSAR has been used to identify collapse hazards and surface-subsidence activity in karst regions [20,21]. Rainfall-infiltration and unsaturated-seepage theories explain how groundwater-level changes reduce soil strength [22,23]. Electrical resistivity imaging helps constrain subsurface heterogeneity, but its interpretation benefits from borehole and geotechnical information [15,24]. Rainfall-related instability also depends on antecedent hydrological conditions, transient pore-pressure response, and changes in soil suction [16,25].

Hydrological forcing does not necessarily produce an immediate deformation response. Field observations document seasonal movement and delayed responses to rainfall [25,26], while hydromechanical models describe feedback between fissures, groundwater fluctuations, and movement [27,28]. These findings motivate combining spatial evidence with appropriately paired temporal observations, without treating non-synchronous datasets as a statistical correlation sample.

Previous studies have established relatively mature methodological systems for landslide identification using InSAR monitoring, interpretation of subsurface structures through karst geological investigations, and slope-stability evaluation by numerical simulation. Nevertheless, three limitations remain for operational high-fill expressway embankments. First, recurrent settlement is often attributed to post-construction compaction or shallow structural distress, with insufficient attention paid to the coupling between natural-slope creep and differential embankment settlement. Second, remote-sensing deformation, borehole water levels, karst cavities, radar-detected voids, and treatment boundaries have rarely been organized into a unified evidence chain. Third, remediation measures have seldom been comparatively evaluated for their ability to control slope-scale creep. To address these limitations, this study makes three contributions using the K610 section of the Rucheng-Chenzhou Expressway as a case study: (1) it establishes a multi-source evidence chain integrating InSAR, ground monitoring, borehole investigation, hydrochemistry, radar detection, construction and maintenance records, and numerical analysis; (2) it demonstrates that the K610 distress is fundamentally differential embankment settlement driven by creep of the natural slope rather than an isolated pavement-structure defect, thereby shifting the remediation concept from pavement maintenance to integrated embankment-slope management; and (3) it constructs a three-dimensional FEM for self-weight, natural, and extreme-rainfall conditions to test the effects of hydrological factors on slope deformation, concentration of distress, and slope stability.

## 2. Study Area and Evolution of Distress

### 2.1. Engineering Geological Setting

The distressed K610 section is located on the Rucheng-Chenzhou segment of the G76 Xiamen-Chengdu Expressway in Yizhang County, Chenzhou City, Hunan Province (Figure 1), and extends from K610+000 to K610+145. This four-lane expressway has a design speed of 80 km/h and an embankment width of 24.5 m and was opened to traffic in December 2012. The distressed section occupies a fill embankment and a cut-fill transition. The original terrain is higher in the south and lower in the north; fill thickness is approximately 10–16 m on the northern side and much smaller on the southern side, forming a typical asymmetric high-fill embankment on a steep slope.

Geological records from the construction stage indicate that the section lies in a locally developed karst-dissolution zone and that the upper soils consist mainly of weakly expansive silty clay. The original design used benched excavation and geogrid reinforcement to treat the cut-fill transition and steep-slope embankment. Persistent hillslope seepage and localized soft ground were encountered during construction, prompting the addition of subsurface drainage trenches, a median drainage ditch, a graded-crushed-stone cushion, and soft-soil replacement. These records indicate that hydrogeological conditions and nonuniform fill thickness were important pre-existing controls before the road entered service.

### 2.2. Evolution of Distress and Previous Remedial Measures

The operational distress has been persistent and recurrent (Table 1). Following prolonged rainfall in 2014, with precipitation of 1309.2 mm/yr, the right carriageway from K610+010 to K610+060 experienced 5–7 cm of pavement settlement; the toe drainage ditch on the northern side of the embankment was compressed and cracked, and longitudinal cracks developed in the median. Settlement continued during 2015–2016, and pavement overlays were used primarily to restore traffic serviceability. In 2017, comprehensive remediation was implemented, including subsurface drainage trenches, steel pipe piles, grouting, continuously reinforced concrete slabs, and pavement reconstruction. Although settlement near the end of the distressed section was partially controlled, the central portion did not become fully stable. Pavement subsidence and embankment deformation continued along the right carriageway from K610+010 to K610+060 after 2019, and maintenance records from 2021 to 2024 show annual settlement on the order of centimeters to decimeters. This temporal pattern indicates that the previous treatment altered the local deformation rate but did not eliminate the deep hydrogeological processes governing the evolution of distress.

## 3. Data Sources and Analytical Methods

### 3.1. Multi-Source Dataset

The dataset comprises engineering design documents and as-built change records, special investigation and remediation records from 2017, settlement observations from 2022 to 2024, ground-penetrating radar surveys, fluoride and cadmium measurements of water samples, InSAR deformation maps, a three-dimensional terrain model, and numerical-simulation results (Table 2). The 2017 investigation included 39 boreholes, 14 of which encountered groundwater and 11 of which intersected karst cavities. Three high-density electrical resistivity profiles revealed low-resistivity anomalies beneath the distressed section. The high-density electrical resistivity survey used a Wenner array [29], a resistivity meter, and a multi-channel electrode switching unit. These data were organized according to a four-component framework comprising the deformation field, geological structure, hydrological processes, and mechanical response. The datasets were compared for spatial agreement between deformation zones and subsurface anomalies, consistency between the available deformation observations and the documented stages of distress, and compatibility with the mechanisms indicated by the numerical analyses [14].

### 3.2. Quantitative InSAR Deformation Analysis

A time-series deformation analysis was conducted using the small baseline subset synthetic aperture radar interferometry (SBAS-InSAR) method and an ascending-track Sentinel-1A C-band synthetic aperture radar (SAR) image dataset. The images span July 2024 to March 2026, consistent with the time interval of the deformation results presented below. Interferometric Wide-swath (IW) single-look complex (SLC) data were used; the study area lies in subswath IW2, with a nominal incidence-angle range of 33.7–39.1 degrees and a southeast-looking radar line of sight. Processing included image coregistration, interferogram generation, topographic-phase removal, correction of orbital and atmospheric errors, phase unwrapping, and time-series inversion. Stable scatterers were selected to improve temporal reliability along the expressway and on exposed slope surfaces [8,10]. The outputs include mean LOS velocity, cumulative LOS displacement, and time series for representative points. Because only a single orbit was used, the LOS measurements are interpreted as indicators of relative activity and not directly equated with vertical settlement [30,31].

### 3.3. Numerical-Simulation Scheme

Slope stability was evaluated using the three-dimensional finite-element strength reduction method (SRM) [32,33,34,35]. This classical nonlinear numerical approach progressively reduces the shear-strength parameters of the geomaterials, namely cohesion (c) and friction angle (φ), while maintaining the model geometry, boundary conditions, and external loads. Reduction continues until the slope reaches a critical failure state, and the corresponding reduction factor is taken as the safety factor Fs. Unlike conventional limit-equilibrium methods, which require an assumed slip-surface geometry, SRM automatically identifies potential slip surfaces, reproduces progressive slope failure, and characterizes the deformation evolution and plastic-zone coalescence of the coupled slope-embankment system under rainfall conditions. It is therefore well suited to the complex strata and fill embankment at this site. A three-dimensional FEM measuring 315 m east–west, 450 m north–south, and 167 m vertically was constructed from a 1:1000 high-resolution DEM of the study area (Figure 2a). The domain was discretized using a mixed mesh dominated by hexahedral elements. From top to bottom, the strata comprise a silty-clay cover, strongly weathered carbonaceous shale, and moderately weathered limestone bedrock, for a total of 291,007 elements (Figure 2b).

The model base was fully fixed, the normal displacement was constrained on the four lateral boundaries, and the ground surface was free. An ideal elastoplastic Mohr–Coulomb model was adopted. The natural unit weight, saturated unit weight, cohesion, and internal friction angle used in the model were primarily determined from the engineering geological investigation report and laboratory geotechnical test results. The elastic modulus values were estimated using the investigation data, engineering experience with similar geomaterials, and engineering analogy (Table 3). Instability during strength reduction was identified from abrupt displacement at characteristic points and coalescence of the plastic zone. Displacement, shear strain, plastic strain, and the safety factor were calculated for each condition.

The analysis comprised three stages. First, a self-weight equilibrium analysis was used to identify the initial deformation-concentration zone caused by the asymmetric geometry of the high-fill embankment. Second, the safety factor under natural conditions was calculated using the strength reduction method. Finally, strength reduction analysis under extreme-rainfall conditions was performed to quantify the weakening effect of water on slope stability. As a reference for extreme-rainfall conditions, the Severe Weather Information Centre (SWIC) of the World Meteorological Organization (WMO) uses a reporting threshold of at least 50 mm of precipitation in the preceding 24 h [36]. Rainfall thresholds for landslide initiation can also depend on antecedent hydrological conditions [37]. The groundwater table under extreme-rainfall conditions was estimated from regional historical rainfall information and the geological and hydrological data for the study area. It was used as an input to the finite-element model to indirectly account for changes in groundwater levels caused by rainfall infiltration and their effects on slope stability. Stability was then evaluated using the strength reduction method. The simulation results were spatially compared with the ground-settlement zone, InSAR anomaly, and groundwater-rich zones to test the hypothesized mechanism of rainfall infiltration, water-induced weakening, slope creep, and differential embankment settlement.

## 4. Results of the Multi-Source Evidence Analysis

### 4.1. Slope-Scale Deformation Identified by InSAR

The InSAR interpretation in Figure 3 shows that deformation at the K610 section of the Rucheng-Chenzhou Expressway is not confined to the pavement or to a single fill body. Anomalous deformation points are distributed mainly along the high-fill side of the embankment, the slope surface, and the slope toe. Local orange-yellow points correspond to deformation features delineated in the field, indicating that the embankment is not undergoing isolated point settlement but lies within a broader deformation zone on the natural slope. Mean LOS velocities range from −30 to −8 mm/yr, with the largest negative velocities concentrated on the northern high-fill embankment and the adjacent slope. This spatial pattern strongly suggests coupling between embankment distress and slope creep.

To evaluate the persistence of the deformation anomaly, cumulative LOS displacement time series were analyzed for nine representative points identified in Figure 3. As shown in Figure 4, points P1-P3 exhibit persistent negative cumulative displacement from July 2024 to March 2026, reaching approximately −40 mm by the end of the observation period. All three curves show a marked acceleration during the 2025 rainy season that coincides temporally with heavy rainfall, suggesting that changes in slope moisture may trigger or amplify deformation [16,38,39].

The time series for points P4-P9 exhibit a similar staged pattern of slow initial accumulation, acceleration during the rainy season, and subsequent deceleration. Final cumulative displacement varies among the points, reaching approximately −50 mm at some locations, which is broadly consistent with the spatial distribution of pavement settlement on the northern side of the study section. The strongly synchronous deformation histories further exclude incidental effects such as noise at individual scatterers or localized movement of structures. Instead, they indicate that rainfall infiltration causes water-induced strength degradation of the geomaterials, producing a system-scale progressive creep response of the coupled slope-embankment system with coherent spatial and temporal characteristics.

### 4.2. Geological and Hydrogeological Controls

Distress at the K610 section is controlled first by the geometric asymmetry of the steep-slope high-fill embankment. The original terrain is higher on the left and lower on the right, and the combined thickness of embankment fill and the original soil cover reaches approximately 16 m. Variations in fill thickness create the geometric conditions for nonuniform settlement between the two carriageways and across each carriageway. The 2017 investigation identified karst cavities in 11 boreholes, corresponding to a cavity-encounter rate of approximately 28.2% and a mean linear karstification rate of approximately 8.2%. The cavities occur at depths of 9–19.2 m, with heights of 0.9–2.3 m and a mean height of 1.68 m. Groundwater is shallow, with depths to water of 5.0–8.8 m, and both cavity water and groundwater generally flow from south to north. Three high-density electrical resistivity profiles, totaling approximately 900 m in length, were completed using a 5 m electrode spacing. Profiles A–A′ and B–B′ are located, respectively, south of the expressway and within the central median, and both were surveyed from west to east. Profile C–C′ lies north of and approximately perpendicular to the expressway and was surveyed from south to north (Figure 5).

The results are shown in Figure 6. Apparent resistivity in the underlying strata ranges from 1 to 471 Ω·m and varies markedly both laterally and vertically. Extensive low-apparent-resistivity zones in the lower strata may represent water-rich zones, clay-rich karst infill, or their combined effects [24,40,41]. Comparison of profiles A–A′ and B–B′ further shows a low-resistivity anomaly beneath the most severely distressed portion of the embankment. This anomaly is interpreted as a subsurface flow pathway through which groundwater migrates from south to north and converges near the slope toe, where it emerges as springs.

Five water samples were collected along the study section to investigate the relationships among water in the distressed zone, the tailings reservoir, surface water, and shallow groundwater; the sampling locations are shown in Figure 5. The samples were analyzed primarily for fluoride and cadmium. As shown in Table 4, fluoride concentrations differ markedly among the samples. The concentration is 17.54 mg/L in the tailings reservoir, 0.14 mg/L in the hilltop reservoir south of the embankment, and 0.43–0.85 mg/L, with a mean of 0.59 mg/L, in the three samples from the distressed zone. The order-of-magnitude difference between fluoride concentrations in the distressed-zone samples and the tailings reservoir excludes the tailings reservoir as the principal source of groundwater recharge. The dominant water process inducing embankment subsidence is instead the convergence beneath the embankment of surface runoff and shallow groundwater generated by rainfall and infiltration on the southern hillslope. This interpretation is informed by studies of rainfall infiltration, pore-pressure response, and partially saturated soil behavior in slope instability [3,42,43].

### 4.3. Numerical Evaluation of the Proposed Mechanism

The three-dimensional numerical simulations were used primarily to determine whether the plastic zones, shear-strain concentration zones, and maximum-displacement zones coincide with the InSAR-active area, ground-settlement section, and radar-detected voided section, thereby testing the proposed evidence chain of rainfall infiltration, water-induced weakening, slope creep, and differential embankment settlement.

#### 4.3.1. Simulation Results Under Self-Weight

Figure 7 shows the displacement and strain distributions under self-weight, with only gravitational loading considered. Total displacement is concentrated mainly in the northern fill zone and the middle to lower part of the slope. The maximum total displacement, 0.347 m, occurs near the toe of the fill embankment. Displacement generally increases downslope from south to north and from left to right. The *Y*-direction displacement has a distribution similar to that of total displacement, with relatively large horizontal movement in the embankment and near the slope toe. The *Z*-direction displacement (settlement) is likewise concentrated in the northern high-fill embankment and the underlying silty-clay cover. These results show that the asymmetric fill geometry creates an inherent predisposition to differential settlement and provides the geological precondition for subsequent rainfall-induced distress.

#### 4.3.2. SRM Strength-Reduction Analysis Under Natural Conditions

Slope stability under natural conditions was evaluated using the strength reduction method (SRM). The calculated safety factor is Fs = 1.12, indicating that the slope is marginally stable under natural conditions but has a limited safety margin. The SRM results for slope failure under natural conditions are shown in Figure 8.

Under natural conditions, both maximum shear strain and effective plastic strain are concentrated primarily in the northern fill zone, and the zone of high shear strain has the greatest risk of sliding deformation and shear failure. Maximum displacement is likewise concentrated in the northern embankment fill and is spatially consistent with the InSAR deformation anomaly. The total-displacement vector field indicates that deformation is directed generally toward the northern side of the expressway, broadly matching the sliding direction inferred in the field and further confirming that embankment deformation is controlled by creep of the natural slope. Field monitoring indicates slow creep settlement and gradual development of embankment distress; the finite-element results are therefore consistent with the long-term maintenance records and the time-series deformation pattern.

#### 4.3.3. SRM Strength-Reduction Analysis Under Extreme-Rainfall Conditions

Under extreme-rainfall conditions, sustained infiltration rapidly raises the groundwater level, while the associated increase in pore-water pressure reduces effective stress and markedly decreases the shear strength of the geomaterials. The interpretation draws on studies of unsaturated shear strength, transient seepage, and unsaturated hydraulic properties [22,23,44,45]. Strength-reduction analysis yields a safety factor of Fs = 1.01, indicating that the slope approaches a critical state of instability under extreme rainfall. The results are shown in Figure 9.

Rainfall causes slope deformation to increase rapidly and the maximum-displacement zone to expand into the slope interior. Under extreme-rainfall conditions, the shear-strain concentration zone extends rapidly from the slope crest to the toe and forms a continuous high-shear-strain band, indicating that the potential slip surface is approaching full coalescence. The effective-plastic-strain concentration zone closely coincides with the shear-strain concentration zone. This pattern shows that rainfall infiltration causes widespread shear yielding in the shallow geomaterials and that plastic zones develop and coalesce through the embankment fill and underlying cover, ultimately forming a continuous slip surface. Previous studies have shown that rainfall infiltration and associated pore-pressure changes can reduce slope stability [46,47].

Comparison of the displacement, shear-strain, and plastic-strain contours under natural and extreme-rainfall conditions shows that, when the slope reaches limiting instability, the magnitudes and spatial distributions of maximum displacement, maximum shear strain, and maximum plastic strain are broadly similar under both conditions, and the overall failure mode is shear failure. Rainfall infiltration therefore degrades the mechanical properties of the geomaterials without changing the inherent deformation pattern or failure mechanism. The key difference between the two conditions lies in the available stability margin: the safety factor under extreme rainfall is 9.8% lower than that under natural conditions. During extreme rainfall, a shear-slip band connecting the embankment fill with the underlying cover develops rapidly, and the affected area expands markedly, validating the progressive failure mechanism of rainfall infiltration, water-induced weakening, slope creep, and differential embankment settlement. The safety factor approaches 1.0 under extreme rainfall, indicating a severely depleted stability margin and a substantial risk of failure. These results are consistent with the rapid pavement settlement, longitudinal cracking, and compression-induced cracking of drainage facilities observed after the 2014 heavy rainfall and provide mechanical support for the interpretation of rainfall-related slope instability and concentrated embankment distress.

#### 4.3.4. Comparison of Simulations Under Multiple Conditions

Taken together, the finite-element results under self-weight, natural, and extreme-rainfall conditions provide mechanical support for the progressive failure pathway revealed by the multi-source evidence chain. Distress at the K610 section of the Rucheng-Chenzhou Expressway is fundamentally the progressive failure of a coupled slope-embankment system dominated by water-induced weakening of the geomaterials, rather than localized damage caused by an isolated pavement-structure defect. The slope safety factor is 1.12 under natural conditions and decreases to 1.01 under extreme-rainfall conditions, demonstrating a pronounced reduction in stability. This pattern is highly consistent with the rainfall response during accelerated slope displacement in the InSAR time series and with engineering records of the first large-scale pavement settlement following heavy rainfall in 2014. Rainfall-induced strength degradation is therefore the fundamental trigger of slope deformation and concentrated distress. This interpretation is consistent with established rainfall-induced landslide mechanisms [3,42,48].

The emergency measures implemented in 2017, including steel pipe-pile reinforcement, grouting, and shallow drainage, locally reinforced and diverted water only within the shallow deformation zone of the embankment. They did not eliminate key deep-seated hazards such as karst-controlled slip zones and groundwater bypass-flow pathways. Under long-term cyclic infiltration from intense rainfall and repeated groundwater recharge, water-induced degradation of the geomaterials persists, residual slope deformation remains active, and instability is still possible during extreme rainfall, consistent with previously reported rainfall- and groundwater-driven embankment and slope instability mechanisms [5,42]. The simulation results agree closely with the recurrent and progressively worsening field distress observed after 2019, confirming the limitations of shallow treatment measures for eliminating water-induced creep-type embankment landslides.

Overall, the finite-element results form a coherent, mutually corroborating evidence chain with the InSAR deformation monitoring, borehole investigations, and high-density electrical resistivity imaging. Together, these data clearly delineate the complete hazard-evolution sequence of rainfall infiltration, water-induced weakening, slope creep, and differential embankment settlement, and systematically clarify the mechanism of water-induced creep distress in high-fill mountain expressway embankments. The findings provide a reliable theoretical and technical basis for risk prediction, mechanistic diagnosis, and fundamental remediation of similar hillside embankments in karst regions.

## 5. Discussion

### 5.1. Multi-Source Evidence Chain for the Landslide Mechanism at the K610 Section

The K610 distress can be conceptualized as a progressive sequence of rainfall infiltration, water-induced weakening, slope creep, and differential embankment settlement (Figure 10), consistent with the roles of infiltration, preferential flow, and groundwater response described in previous studies [3,27,49]. This interpretation is not inferred from any single dataset but is jointly constrained by four categories of evidence.

(1) Deformation-field evidence: the InSAR anomaly extends beyond the embankment itself and is continuous at the slope scale; representative time series show persistent accumulation and rainy-season acceleration and correspond to the ground-settlement section. Previous studies link slow displacement to infrastructure damage [50,51] and combine InSAR with geological and geotechnical evidence to interpret progressive slope deformation [52]. (2) Geological-structure evidence: boreholes intersect karst cavities and shallow groundwater, and low-resistivity anomalies indicate possible water-rich or weak zones beneath the distressed section. This interpretation is informed by studies of electrical-resistivity imaging and moisture dynamics in landslides [40,41,53]. (3) Hydrological and shallow-subsurface evidence: hydrochemical results show that the tailings reservoir is not the dominant groundwater-recharge source and that rainfall infiltration is the primary recharge mechanism, while radar-detected voids record the shallow response of deep deformation within the pavement structure. (4) Mechanical evidence: the safety factor decreases from 1.12 under natural conditions to 1.01 under extreme rainfall, and the shear-strain concentration zone broadly overlaps the observed active area. The four categories of evidence are broadly consistent in spatial location, temporal evolution, and physical mechanism. Together, they support a creep mechanism involving the coupled slope-embankment system [26,27,28] and reduce the risk of misdiagnosis based solely on pavement settlement or a single remote-sensing image.

### 5.2. Comparison with Similar Studies

Table 5 compares the K610 case with representative recent studies in terms of study scale, data sources, core methods, dominant controls, and key findings. Fan et al. [14] combined InSAR-based displacement analysis, geophysical inversion, and numerical simulation to assess risks from a translational highway landslide. Jiang et al. [54] investigated slope deformation associated with reservoir water-level fluctuations using InSAR and numerical simulation. At a regional scale, Li et al. [55] examined the spatial distribution and conditioning factors of highway geohazards, whereas Zhang et al. [56] used SBAS-InSAR to identify and assess potential landslides in the Xiaojiang River Basin. Peduto et al. [7] further linked multitemporal road-damage records with landslide-induced displacement for quantitative risk analysis. In contrast to these respective objectives, the K610 case focuses on recurrent settlement of a high-fill embankment in karst terrain and integrates construction and remediation records, deformation observations, subsurface investigations, hydrochemistry, and mechanical analysis to examine the relationship between embankment distress and natural-slope movement.

### 5.3. Evidence Limitations and Implications for Remediation

The proposed analytical framework is subject to four evidence-related limitations. (1) Only single-track InSAR data were used, so LOS displacement cannot be uniquely decomposed into vertical and slope-parallel components; atmospheric delay, vegetation coherence, and reference-point selection may also affect velocity estimates [13,31,57]. (2) The hydrochemical dataset comprises only five samples and a single diagnostic ion, which is insufficient to quantify mixing ratios and recharge pathways among different water sources. (3) The material parameters and hydraulic boundaries of the model lack in situ testing and sensitivity analysis. (4) The borehole and groundwater information, settlement records, InSAR time series, hydrochemical samples, and finite-element results differ in observation period, spatial location, and sampling structure. They do not form a consistently paired dataset for an all-variable correlation matrix. In particular, complete synchronous observations of precipitation, groundwater levels, infiltration, pore-water pressure, effective stress, and geomaterial shear strength are unavailable. Accordingly, no statistical correlation analysis among all these variables was performed, and the evidence chain represents spatial and mechanistic consistency rather than demonstrated statistical dependence. Future work should combine synchronous monitoring and geotechnical tests, align comparable observations in space and time, and evaluate correlations and lagged responses for appropriately paired variables [25,41].

Despite these limitations, the K610 case shows that the treatment unit for recurrent distress in high-fill embankments in karst mountain areas should be expanded from the pavement and local embankment to the integrated natural slope-high-fill embankment-groundwater system. Specifically: (1) interception and drainage measures should account for effective groundwater-level control and deep karst bypass-flow pathways, because shallow subsurface drains alone cannot eliminate high pore pressures and internal erosion within the slope; (2) load-bearing structures should penetrate karst-weakened zones and socket into a competent bearing stratum, otherwise progressive failure may persist through pile-soil interaction; (3) remediation should cover the full width of both carriageways rather than only the most severely distressed segment, thereby reducing the risk of recurrence in adjacent sections after stress redistribution; and (4) monitoring should integrate InSAR, GNSS/leveling, deep displacement, rainfall, and groundwater levels to enable dynamic evaluation of treatment effectiveness and residual risk [12,15,58].

## 6. Conclusions

This study focuses on settlement disasters caused by creep landslides of subgrades on mountainous expressways. By integrating multi-dimensional data including InSAR monitoring, field investigation, hydrochemical tests and 3D numerical simulations, a multi-source evidence chain is established to systematically reveal the catastrophic deformation mechanism of coupled slope-subgrade systems. The main conclusions are presented as follows:

1. The time-series InSAR anomaly, field-inferred sliding direction, ground-settlement section, radar-detected voids, and groundwater-discharge direction are spatially consistent. Together, they indicate that pavement subsidence at K610 is the shallow expression of differential embankment settlement driven by slow deformation of the natural slope rather than simple operational-stage compaction.

2. The multi-source investigation identifies three principal controls: the asymmetric high-fill geometry on a steep slope, softening of the geomaterials caused by karst development and shallow groundwater, and a mismatch between the extent of previous treatment and the slope-scale active zone. Fluoride concentrations in water samples from the distressed zone differ markedly from those in the tailings reservoir but are similar to those in surface water on the mountain slope. The tailings reservoir is therefore not the principal groundwater-recharge source, and rainfall infiltration is the dominant recharge pathway in the study area.

3. Three-dimensional strength-reduction analysis yields factors of safety of 1.12 under natural conditions and 1.01 under extreme-rainfall conditions, a decrease of approximately 9.8%. Under extreme rainfall, the shear-strain and plastic zones become increasingly connected and concentrate toward the core distressed section. These results provide mechanical support for the interpretation that groundwater activity reduces slope stability and induces coupled slope-embankment deformation.

4. This study develops a multi-source evidence-chain framework consisting of slope-scale identification using InSAR, analysis of geological and hydrological controls, numerical testing under multiple conditions, and conceptualization of the progressive failure mechanism. The framework can be used to diagnose recurrent embankment distress in karst mountain areas and to define remediation boundaries; its quantitative thresholds and broader applicability require further validation using multiple case studies and long-term monitoring data.

Future research should combine synchronous monitoring of rainfall, groundwater levels, pore-water pressure, and slope deformation with geotechnical tests to establish paired datasets on common spatial and temporal scales. Correlation and lag-response analyses should then be used to examine relationships among the monitored variables and their links to effective stress and shear strength, thereby testing the proposed deformation mechanism.

## Figures and Tables

**Figure 1:**
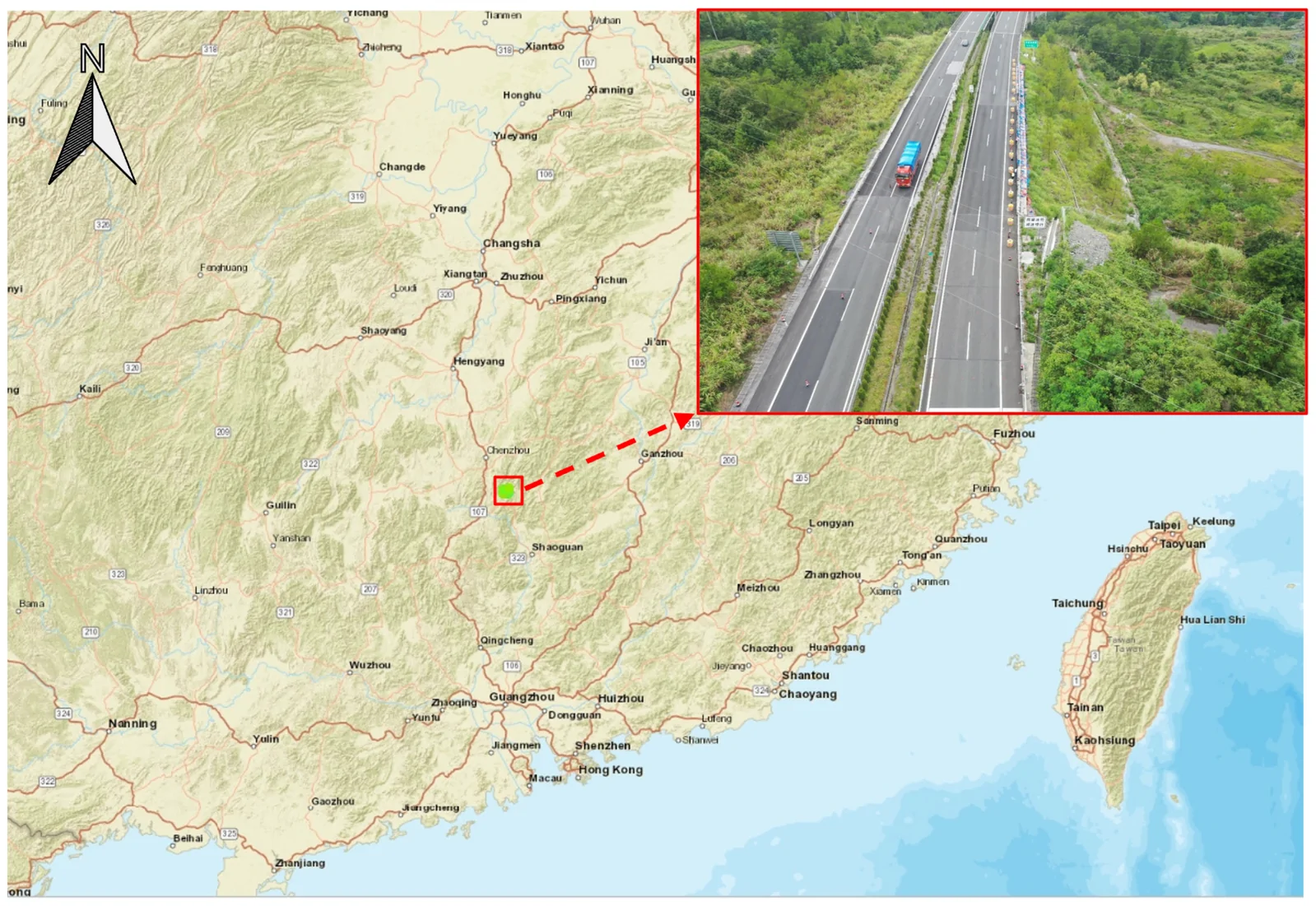
Location of the distressed embankment section and field photographs.

**Figure 2:**
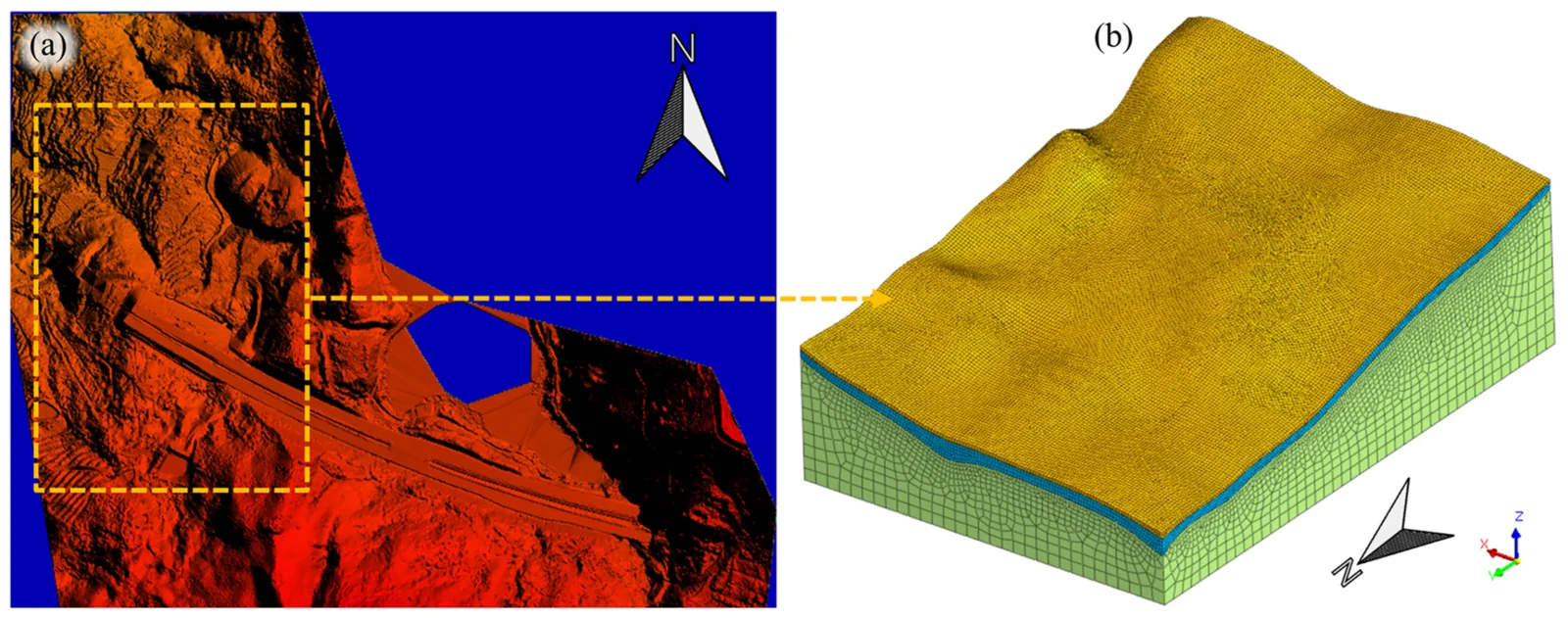
Three-dimensional DEM and FEM: (**a**) DEM; (**b**) FEM.

**Figure 3:**
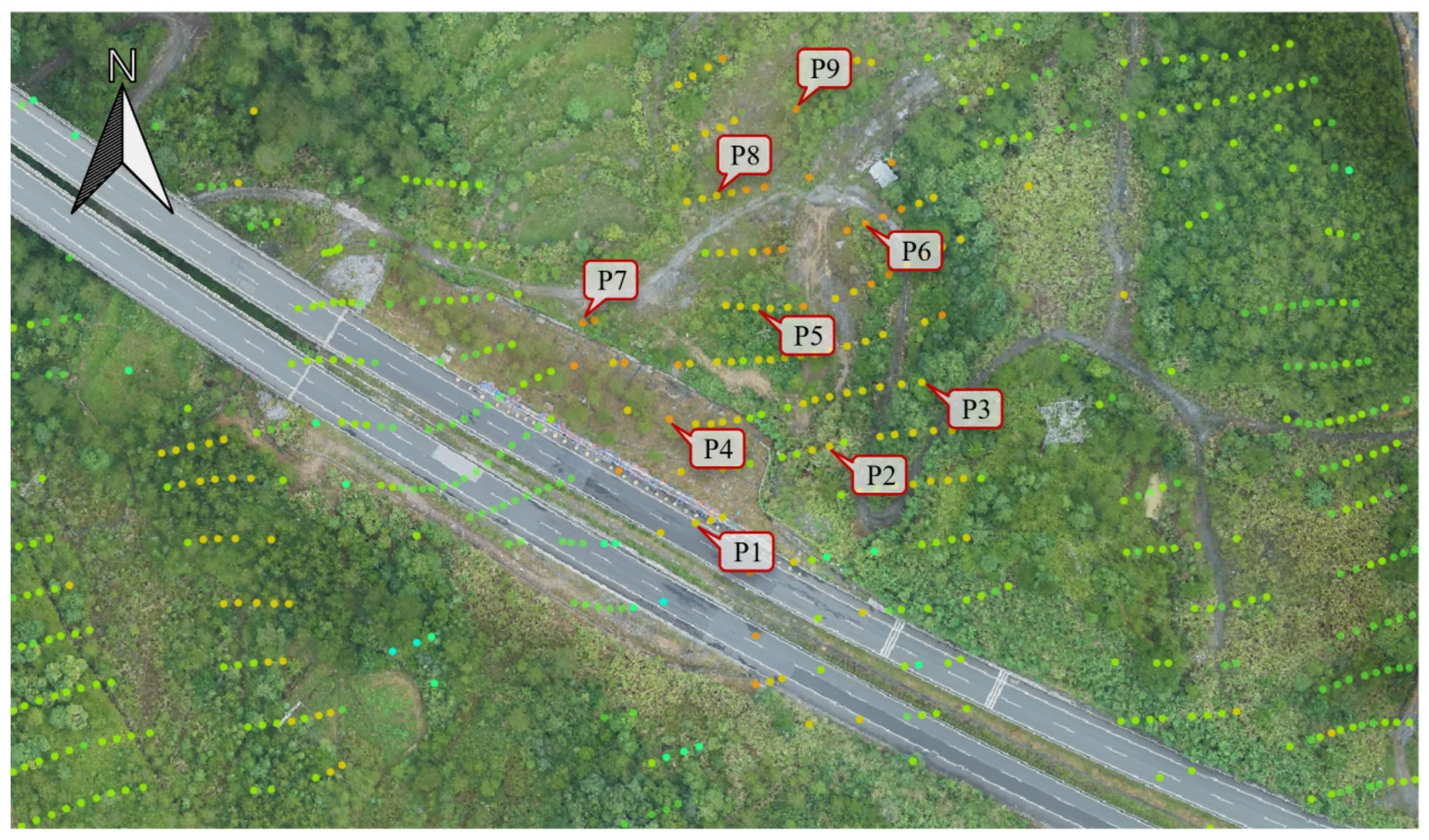
Spatial distribution of InSAR-derived points and locations of representative deformation points at the K610 section.

**Figure 4:**
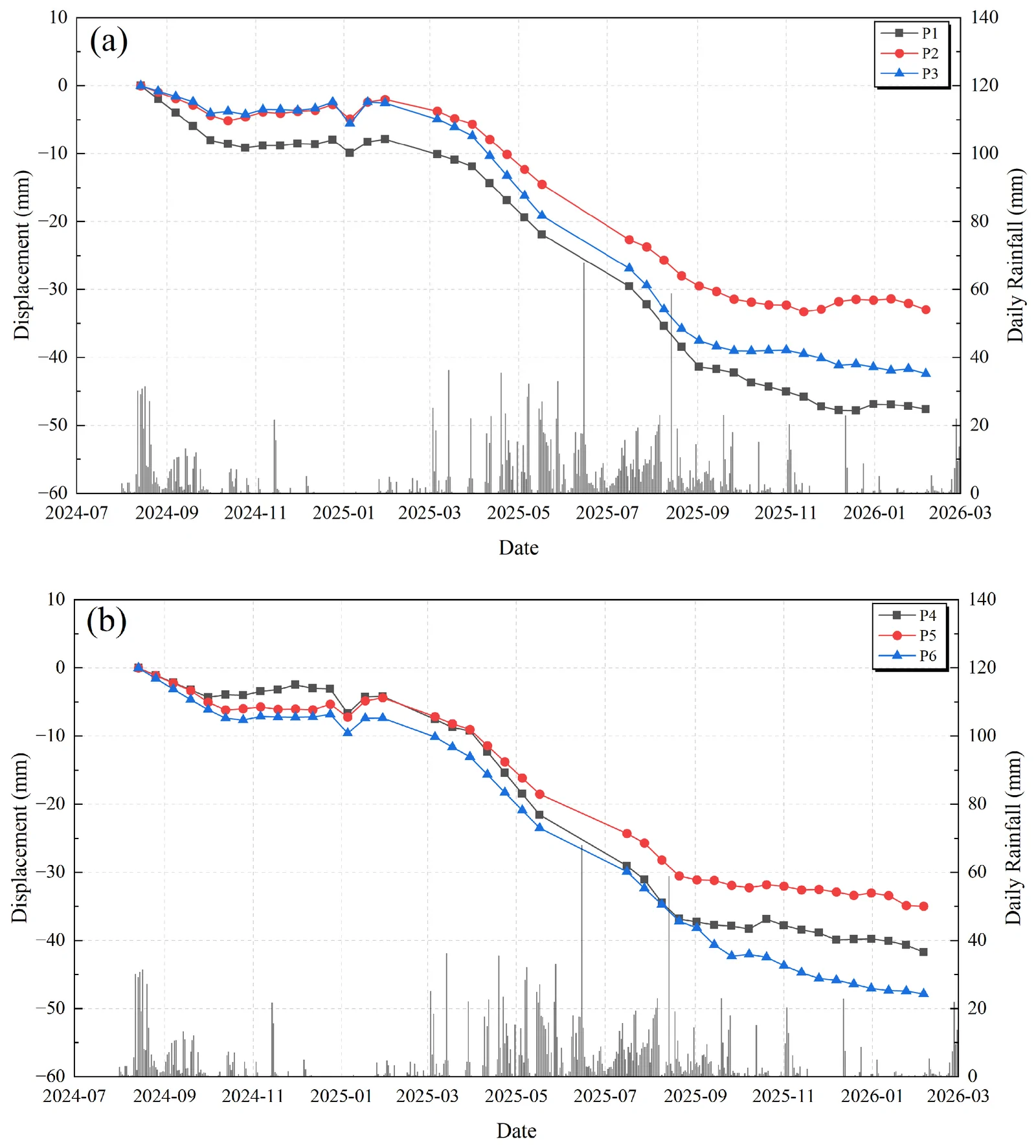

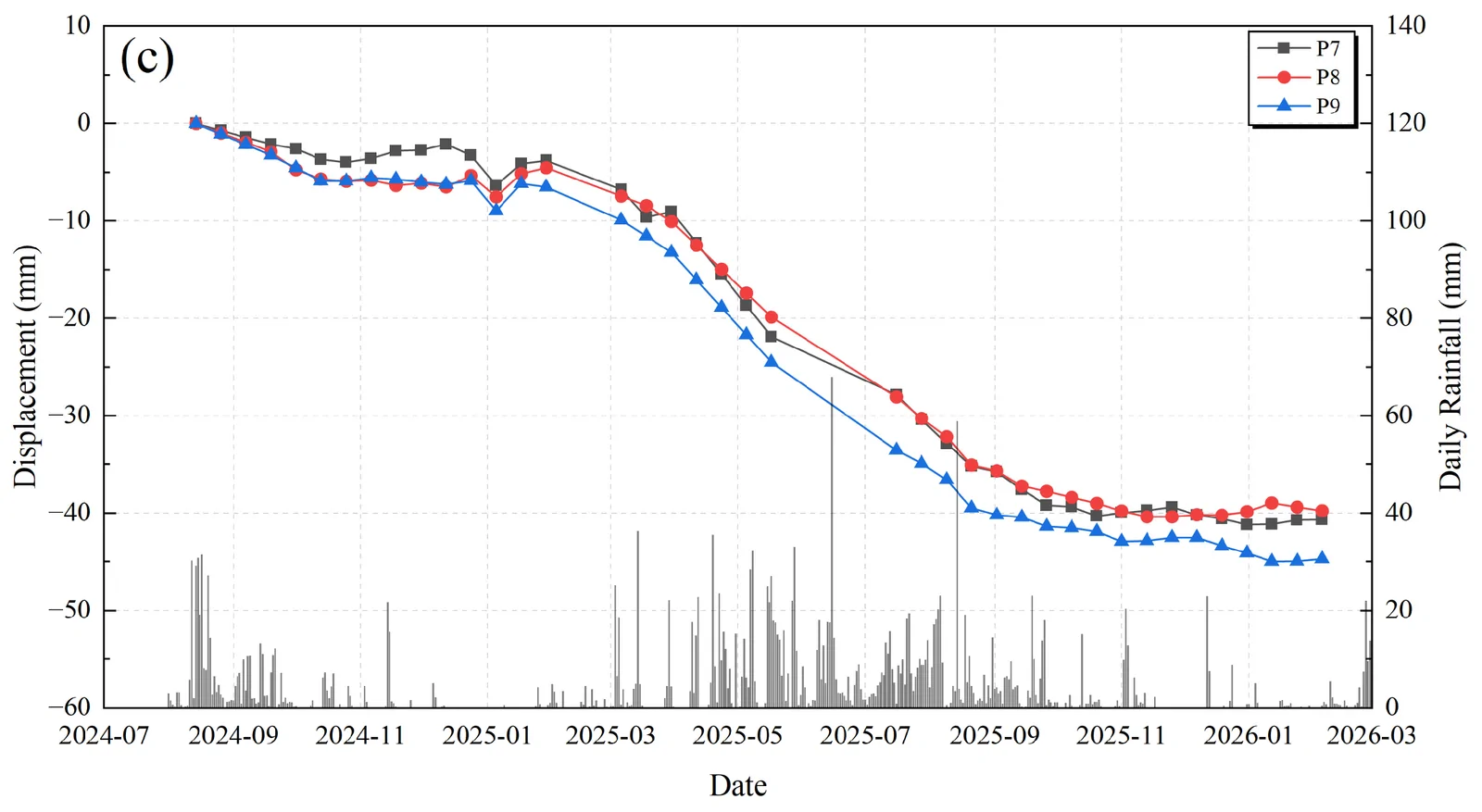
Cumulative LOS displacement and rainfall time series for representative InSAR deformation points at the K610 section: (**a**) P1–P3; (**b**) P4–P6; (**c**) P7–P9.

**Figure 5:**
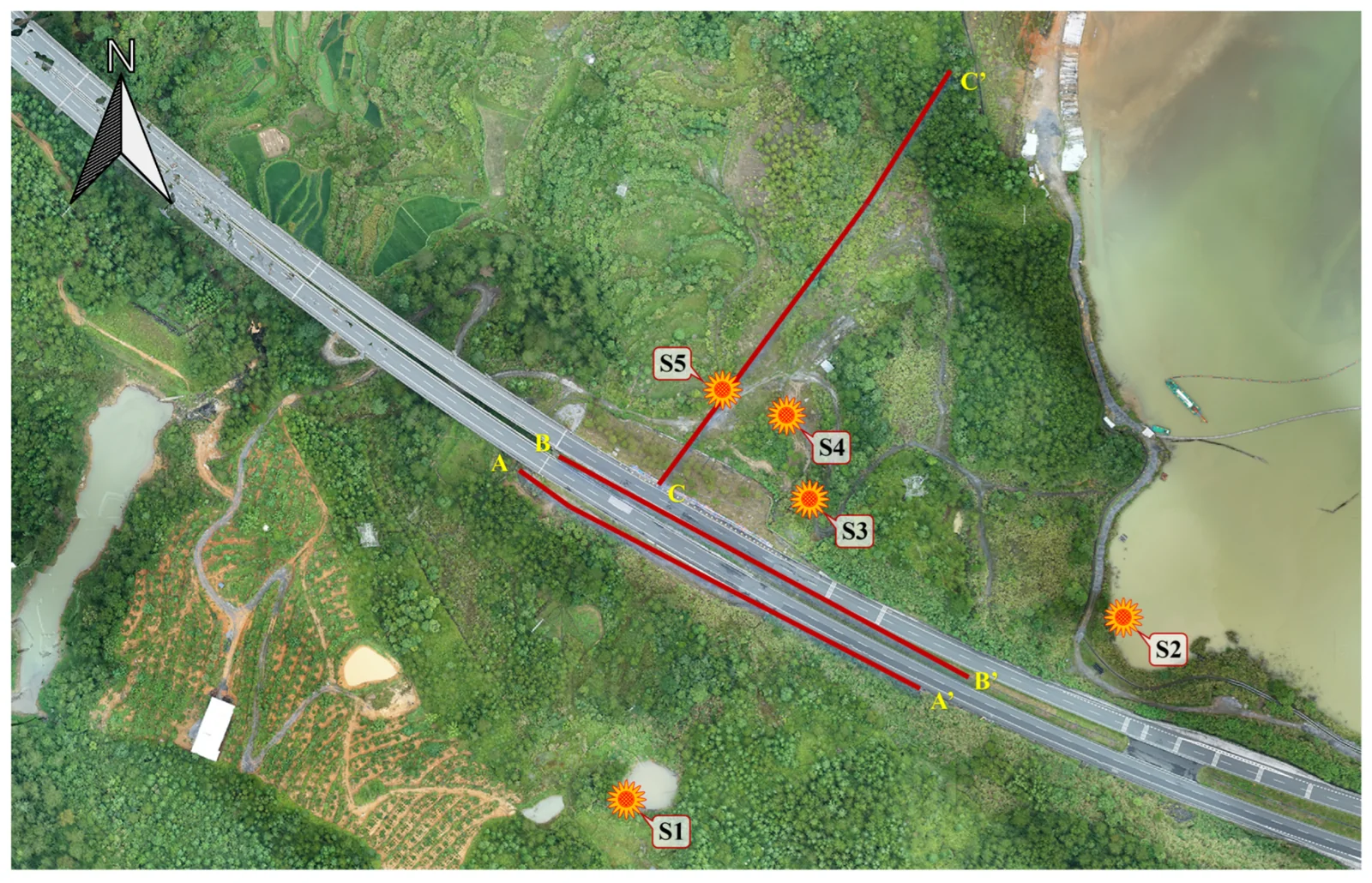
Layout of the geophysical survey lines and water-sampling locations: A–A′, B–B′, and C–C′ denote the three electrical resistivity profiles; S1–S5 denote the water-sampling sites.

**Figure 6:**
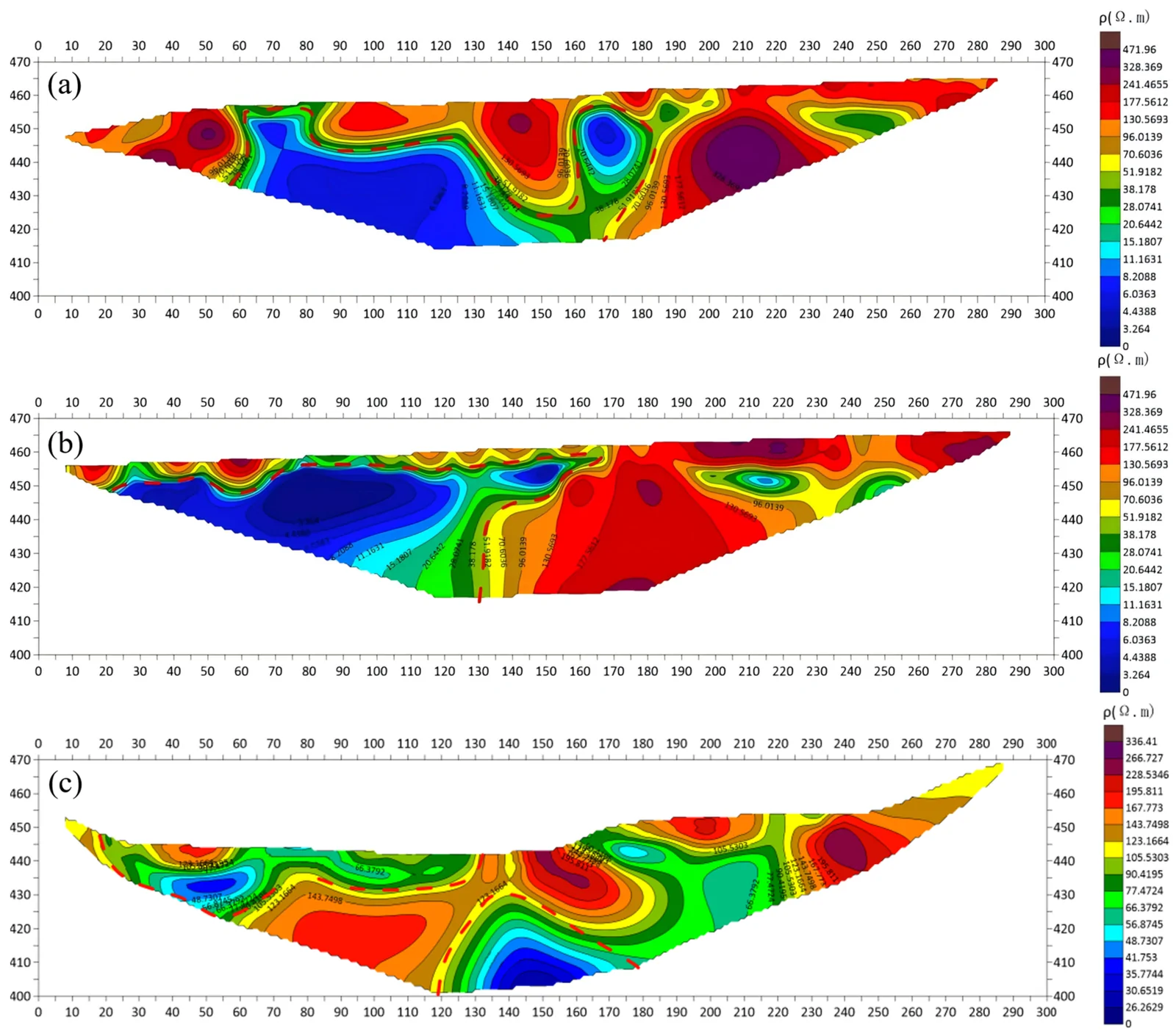
High-density resistivity profiles: (**a**) A–A′ profile; (**b**) B–B′ profile; (**c**) C–C′ profile.

**Figure 7:**
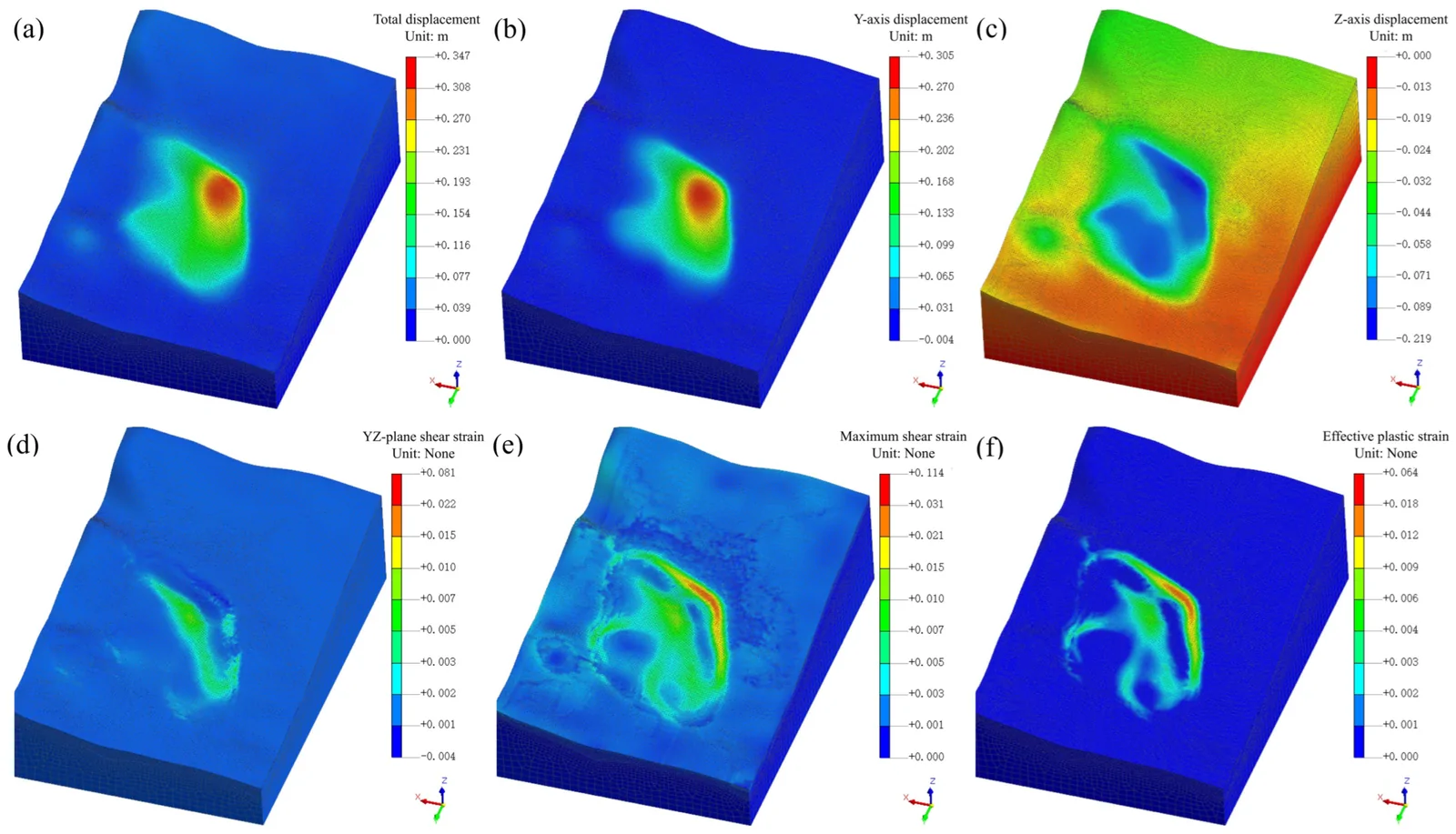
Displacement and strain contours under self-weight: (**a**) total displacement; (**b**) *Y*-direction displacement; (**c**) *Z*-direction displacement; (**d**) shear strain in the YZ plane; (**e**) maximum shear strain; (**f**) effective plastic strain.

**Figure 8:**
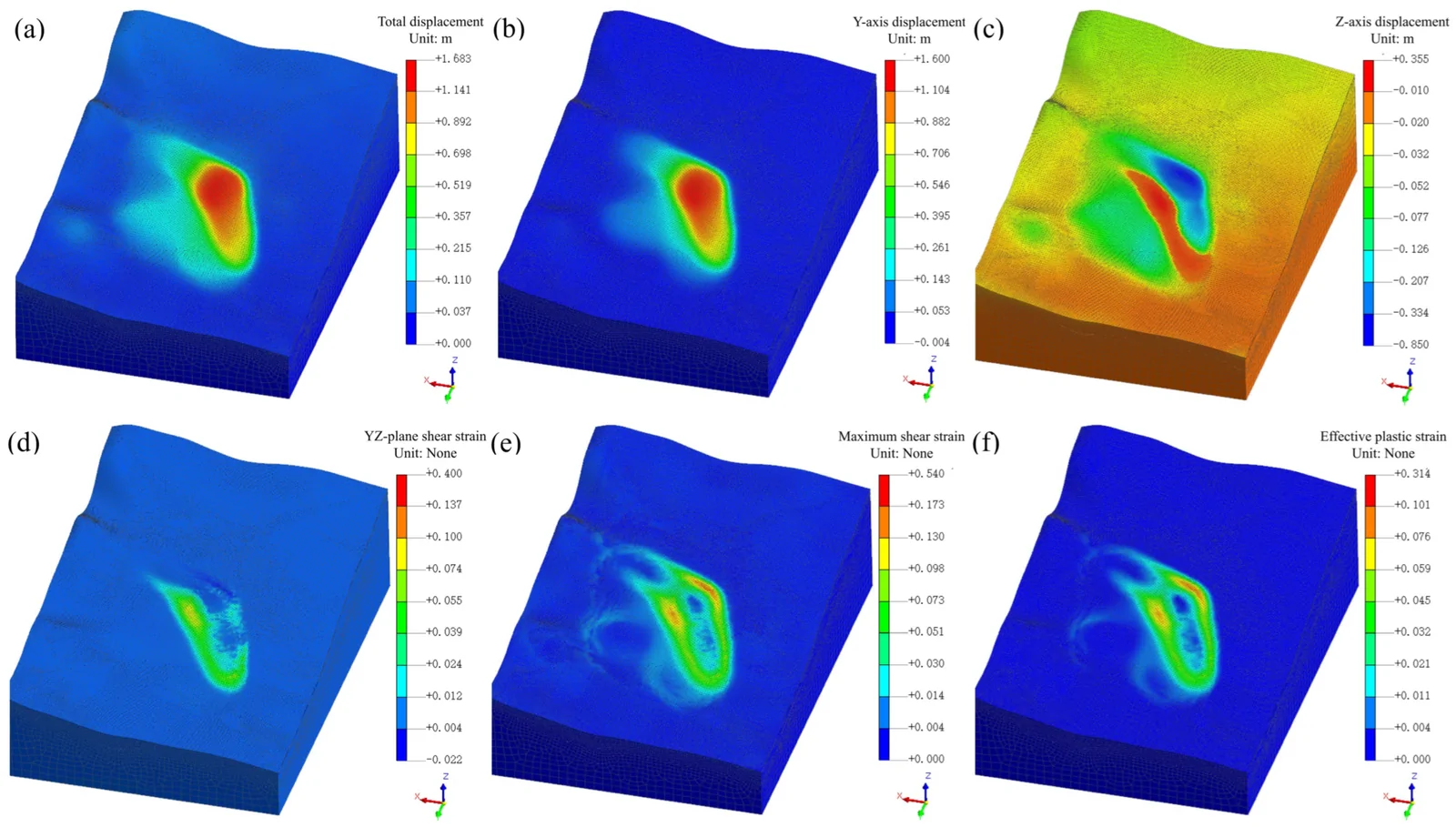
SRM slope displacement and strain contours under natural conditions: (**a**) total displacement; (**b**) *Y*-direction displacement; (**c**) *Z*-direction displacement; (**d**) shear strain in the YZ plane; (**e**) maximum shear strain; (**f**) effective plastic strain.

**Figure 9:**
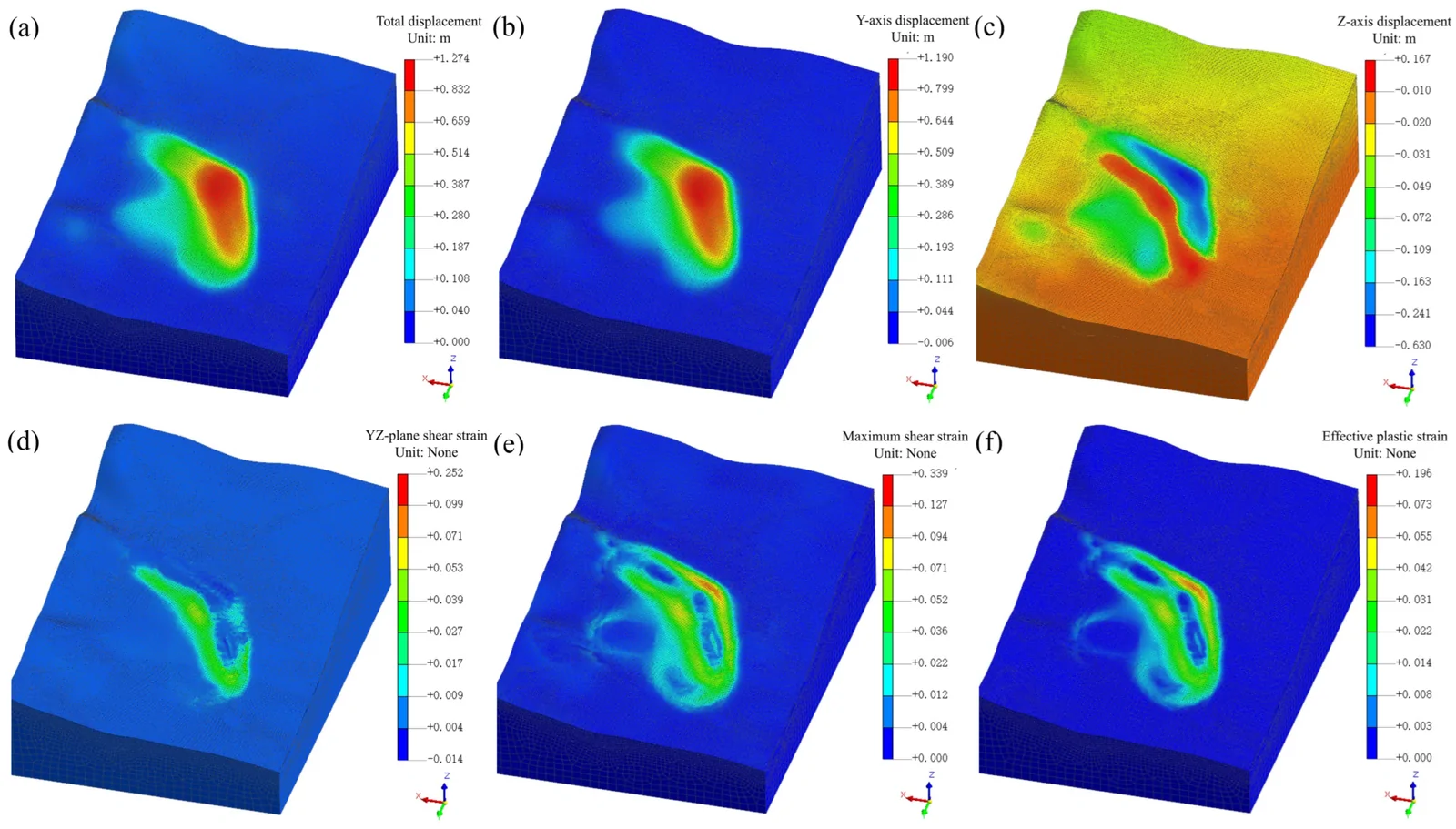
SRM slope displacement and strain contours under extreme-rainfall conditions: (**a**) total displacement; (**b**) *Y*-direction displacement; (**c**) *Z*-direction displacement; (**d**) shear strain in the YZ plane; (**e**) maximum shear strain; (**f**) effective plastic strain.

**Figure 10:**
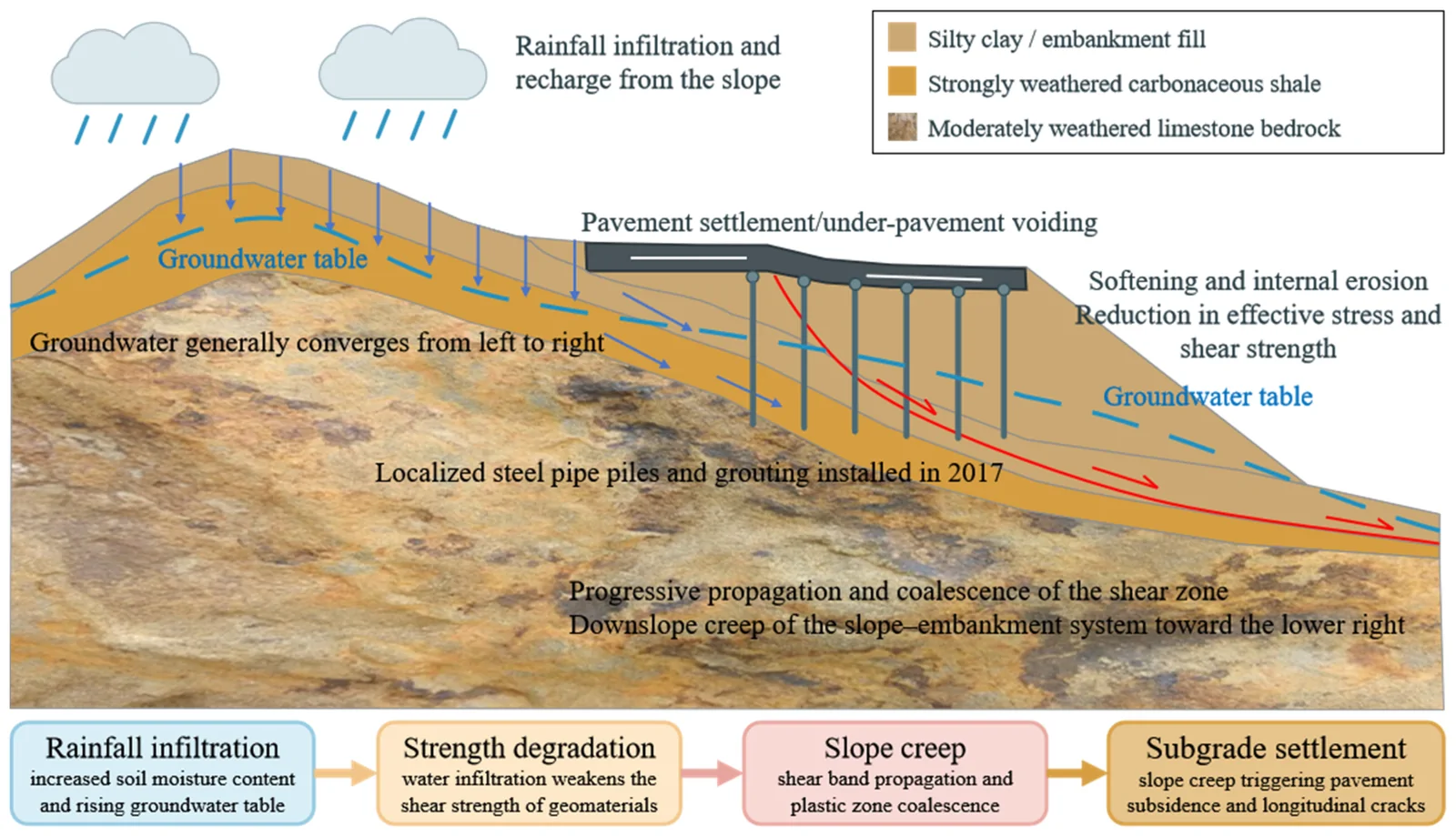
Schematic mechanism of the creep-type embankment landslide at K610.

**Table 1:** Timeline of distress development and remediation at the K610 section.

Period	Embankment Condition and Distress	Mechanistic Implication
2012	The expressway opened to traffic, and the K610 section entered service as a high-fill embankment on a steep slope.	Nonuniform fill thickness and the original slope geometry created the preconditions for subsequent differential settlement and slope shearing.
2014	After rainfall, 5–7 cm of settlement occurred, accompanied by median cracking and compression-induced cracking of the toe drainage ditch.	The distress was coupled with water action and embankment-slope deformation.
2015–2016	Settlement continued, and serviceability was restored mainly by pavement overlays.	Surface rehabilitation did not address the deep-seated source of deformation.
2017	Subsurface drainage trenches, steel pipe piles, grouting, continuously reinforced concrete slabs, and pavement reconstruction were implemented.	Localized reinforcement delayed the distress but did not fully control karst groundwater or slope-scale creep.
2019–2024	Embankment settlement, pavement subsidence, cracking, and damage to drainage facilities continued.	The persistent and progressive distress should be interpreted as deformation of the coupled slope-embankment system.

**Table 2:** Summary of the multi-source datasets and their roles in this study.

Data Type	Data	Role in This Study
InSAR time series	Sentinel-1 C-band SLC data, July 2024–March 2026	Delineate the active-zone boundary and derive LOS velocity, cumulative displacement, and representative point time series.
Ground settlement	Settlement measurements from 2022 to 2024	Verify the correspondence between the InSAR anomaly and the expressway distress zone.
Boreholes and electrical resistivity imaging	39 boreholes, including 14 that encountered groundwater and 11 that intersected cavities, and three high-density electrical resistivity profiles; Wenner array with a resistivity meter and a multi-channel electrode switching unit	Identify fill thickness, cavity locations, groundwater levels, and groundwater-flow direction.
Ground-penetrating radar	Subsidence/voiding beneath the pavement structure, with a maximum interpreted thickness of approximately 36 cm	Reveal the relationship between shallow loosened or voided zones and pavement subsidence.
Hydrochemistry	Fluoride concentrations of 17.54 mg/L in the tailings reservoir, 0.14 mg/L in the hilltop reservoir, and 0.43–0.85 mg/L in samples from the distressed zone	Assess the weak association with external recharge sources or the characteristics of mixed recharge.
Numerical simulation	Three-dimensional terrain and displacement, stress, and strain contours	Evaluate the effects of high groundwater levels, karst-induced weakening, and remediation measures on deformation concentration.

**Table 3:** Physical and mechanical parameters of rock and soil masses.

Stratum Type	γ (kN/m^3^)	γ_sat_ (kN/m^3^)	c (kPa)	φ (°)	E (MPa)
Silty clay	20	22	15	11	16
Strongly weathered carbonaceous shale	24	25	60	26	300
Moderately weathered limestone bedrock	28	29	400	38	6000

**Table 4:** Water-sample analytical results.

Sample ID	Sampling Location	Cadmium (mg/L)	Fluoride (mg/L)	Distance to the Expressway
S1	Tailings reservoir	<0.05	17.54	Approx. 140 m north
S2	Hilltop reservoir	<0.05	0.14	Approx. 40 m south
S3	Ponded water at the toe of the distressed slope	<0.05	0.43	Approx. 50 m north
S4	Ponded water in a surface crack outside the distressed zone	<0.05	0.85	Approx. 80 m north
S5	Groundwater seepage (spring)	<0.05	0.50	Approx. 70 m north

**Table 5:** Systematic comparison of the K610 study with representative related studies.

Study	Study Object	Data Sources	Dominant Controls	Core Research Outputs
Fan et al. [14]	Translational expressway landslide	InSAR + geophysical inversion + numerical simulation	Landslide thickness and volume	Proposed a traffic-loss assessment workflow; focused on estimating the geometry of the active landslide mass
Jiang et al. [54]	Slope deformation induced by reservoir-level fluctuations	InSAR + numerical simulation	Reservoir-level fluctuation as a single forcing factor	Confirmed the control of water-level fluctuations on slope stability under relatively simple hydrological boundary conditions
Li et al. [55]	Distribution of geohazards along expressways in the western Sichuan Plateau	Geohazard inventory + terrain and environmental data	Topography + tectonics + rainfall	Analyzed the spatial distribution and conditioning factors of highway geohazards
Zhang et al. [56]	Potential landslides in the Xiaojiang River Basin	SBAS-InSAR	Rainfall + topography	Identified potential landslides and explained differences in activity; focused on regional screening
K610 section of the Rucheng Expressway (this study)	Creep-type landslide affecting a high-fill mountain expressway embankment	InSAR + boreholes/geophysical investigation + hydrochemistry + radar + construction and maintenance records + numerical simulation	Karst groundwater; fill softening and internal erosion; asymmetric high-fill geometry	Reinterpreted recurrent post-treatment distress as slope-scale creep

## Data Availability

The SRTM DEM is available from https://earthexplorer.usgs.gov/ (accessed on 31 July 2026). The Sentinel-1A data is available from https://search.asf.alaska.edu/ (accessed on 31 July 2026). Other data are contained within the article. Additional information is available upon request to the corresponding author.

## Abbreviations

The following abbreviations are used in this manuscript:

DEM	Digital Elevation Model
InSAR	Interferometric Synthetic Aperture Radar
FEM	Finite Element Model
SRM	Finite Element Strength Reduction Method
